# Erythema nodosum triggered by kerion celsi in pediatrics: literature review and case report^☆^

**DOI:** 10.1016/j.abd.2023.02.009

**Published:** 2023-11-24

**Authors:** Astrid Herzum, Ehab Garibeh, Lodovica Gariazzo, Corrado Occella, Gianmaria Viglizzo

**Affiliations:** Dermatology Unit, IRCCS Giannina Gaslini, Genova, Italy

*Dear Editor,*

Kerion celsi (KC) is a highly inflammatory tinea capitis (TC), occurring predominantly in children of rural areas, and increasingly in urban areas, as pets represent important infection reservoirs.^1^ Causative agents of tinea capitis encompass a great variety of dermatophytes, whose prevalence is geographically influenced: Microsporum canis represents the most common agent in Europe, China and South America; Trichophyton tonsurans in North America and in the UK.^2^, ^3^

The occurrence of erythema nodosum (EN), a septal panniculitis uncommon in children, after KC, can be considered amongst dermatophyte id (dermatophytid) reactions.^4^, ^5^, ^6^ This association was rarely described in literature, especially in children, with only 17 cases reported in English literature in this age group, mainly after Trichophyton *mentagrophytes* scalp infections and mainly after antifungal treatment.^7^, ^8^ Conversely, EN appearing before the administration of antifungal therapy for KC as in the presented case, is unusual, only 5 cases are reported in the literature (Table 1).

**Table 1:** Literature review of pediatric cases of erythema nodosum occurring after Kerion Celsi reported in the English Literature.

1^st^ author	Publication year	Sex	Age (in years)	Aetiologic agent	Time in days from treatment to en development	Therapy	EN healing time after tp (weeks)
Franks	1952	M	9	*T. sulphureum*	Before treatment	Giseofulvin	NR
Smith	1963	M	7	*T. mentagrophytes*	Before treatment	Griseofulvin + topical tioconazole	NR
Stocker	1977	F	12	*T. verrucosum*	Before treatment	Griseofulvin	NR
Martinez-Roig	1982	M	7	*T. mentagrophytes*	7	Griseofulvin + topical potassium permanganate solution	NR
Martinez-Roig	1982	M	6	*T. mentagrophytes*	7	Griseofulvin + topical potassium permanganate solution	NR
Martinez-Roig	1982	M	8	*T. mentagrophytes*	7	Griseofulvin + topical potassium permanganate solution	NR
De las Heras	1991	M	9	*T. mentagrophytes*	before treatment	Griseofulvin + topical tioconazole	6
Calista	2001	F	5	*T. mentagrophytes*	before treatment	Griseofulvin + topical crystal violet	6
Soria	2008	M	9	*T. mentagrophytes*	16	Griseofulvin	NR
Soria	2008	M	11	*T. mentagrophytes*	26	Griseofulvin + Ibuprofen	NR
Bassi	2009	F	8	*T. mentagrophytes*	1	Griseofulvin	6
Zaraa	2012	M	7	Large-spore parasitism	18	Griseofulvin + ciclopiroxolamine cream	12
Castriota	2013	F	9	*T. mentagrophytes*	14	Griseofulvin + topical mupirocin and tioconazole cream + prednisone 1 mg/kg/die	10
Romano	2014	F	4	*T. mentagrophytes*	2	Griseofulvin + topical imidazole	NR
Salah	2021	M	4	*T. mentagrophytes*	20	Griseofulvin	In following days
Salah	2021	M	9	*T. mentagrophytes*	7	Griseofulvin	In following days
Salah	2021	M	14	*T. mentagrophytes*	14	Griseofulvin	In following days

We report the case of a seven-year-old boy who presented an erythematous, tender plaque of the scalp one month prior to the visit, and bilateral painful erythematous nodules of the lower extremities for the past ten days. Topical and oral antibacterial antibiotics were not effective.

During clinical examination a painful occipital plaque was observed (3 × 4 cm), erythematous, with pustules and crust, and loose hair falling out from its exudative surface, combined with occipital lymphadenitis (Fig. 1). On the lower extremities, painful and warm erythematous-violaceous nodules were evidenced, clinically suggestive for EN (Fig. 2).

**Figure 1:**
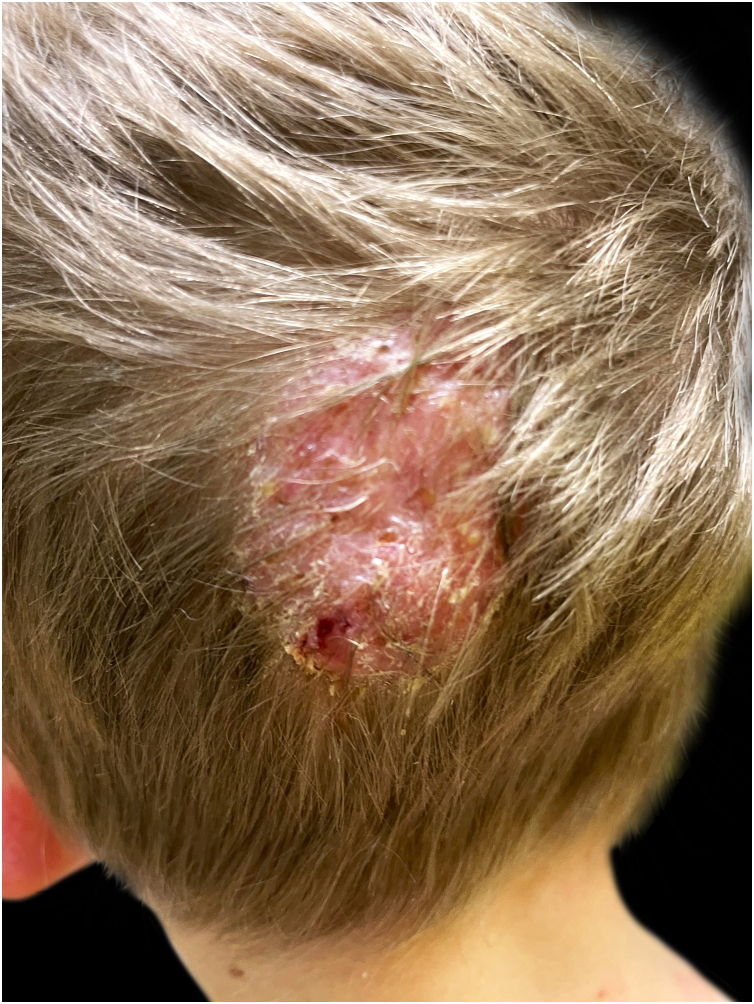
Erythematous purulent and crusted Kerion Celsi of the scalp, with loose hair falling out at the periphery of the lesion.

**Figure 2:**
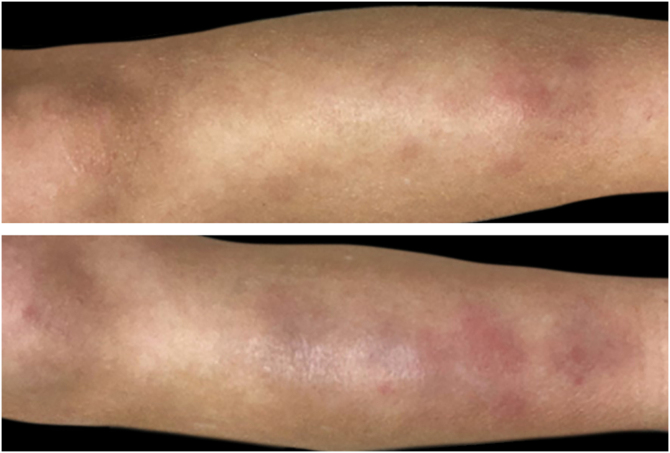
Bilateral erythematous tender nodules of the lower extremities clinically suggestive of erythema nodosum.

Microscopic examination of skin scrapings and hair confirmed the diagnosis of zoophilic dermatophytosis of the scalp caused by *M. canis* (Fig. 3), which was treated with Griseofulvin 250 mg BID (20 mg/kg/day) for 8 weeks, obtaining remission of both conditions, thus confirming the dermatophytid reactive nature of EN of the legs.

**Figure 3:**
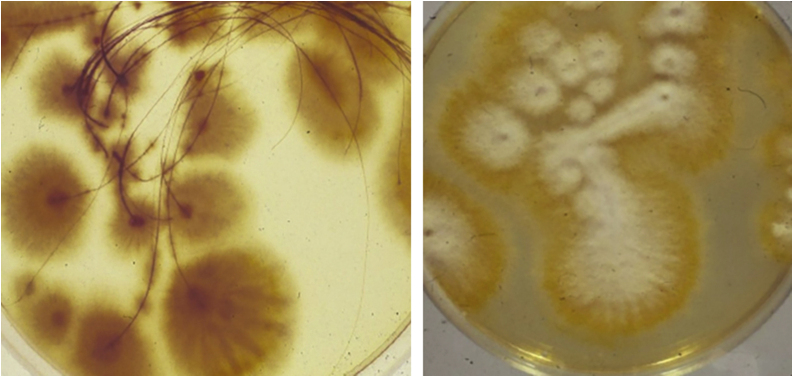
*Microsporum canis* colony growing from hair pulled out at the periphery of the Kerion lesion, forming flat, ivory to white dense cottony colonies.

Id reactions are secondary inflammatory reactions developing from a remote localized immunological insult, such as fungal infections.^4^, ^5^, ^6^ Id reactions possibly exhibit multiple clinical presentations, including localized or widespread vesicular lesions, maculopapular or scarlatiniform eruptions, erythema nodosum, erythema multiforme, erythema annulare centrifugum, Sweet's syndrome, guttate psoriasis, and autoimmune bullous disease.^4^, ^6^

Diagnostic criteria for dermatophytid reaction comprise: (I) A proven dermatophytosis, (II) An eruption in a distant location from fungal infection, and (III) The resolution after antifungal treatment.^7^ On the basis of the abovementioned clinical criteria, we diagnosed clinically an EN-type dermatophytid reaction.

Dermatophytid reactions occur in up to 17% of patients with dermatophyte infections, typically after tinea pedis and, in children, after tinea capitis, mainly presenting as papulo-vesicular eruptions of acral sites and trunk. Also, erythema multiforme, erythema annulare centrifugum, urticarial-manifestations and erythema nodosum, though rarely, have been described.^6^

In literature, EN-type reaction has been mainly described after *T. mentagrophytes* KC (82%) while in the remaining cases Tricophyton sulphureum, Tricophyton verrucosum, and general large-spore parasitism were reported; mean age at onset was 8 years (range 4‒14 years).^7^, ^8^, ^9^, ^10^

In literature, the onset of EN-type reactions after KC is variable, uncommonly (30%) before treatment and more frequently near infection climax (70%), after antifungal administration, occurring meanly 12 days (range 26‒1 days) after antimycotics.^7^, ^8^ Interestingly, the temporal correlation between inflammation-peak and EN, suggests that a phlogosis-induced massive release of auto-antigens may be in play, supporting an autoimmune hypothesis of reactive T-cells, activated by massive antigenic release, induced by the fungal infection.^4^, ^5^, ^6^

Possibly, reactive T-cells, that are activated by antigenic release from a primary stimulus, causing keratinocyte damage, may induce autoimmune-mediated cutaneous phenomena against autologous keratinocyte antigens at distant sites, after lymphocytic dissemination. Indeed, dermatophytid reactions are observed mostly after highly inflammatory forms of dermatophytosis, such as in the presented case, where a great amount of auto-antigens may have been released.^4^, ^5^, ^6^

Of note, the diagnosis of Id reactions is essential for the correct management of the patient, as these autoimmune reactions mainly (70%) occur after antimycotic initiation and can be misdiagnosed with allergic reactions to antifungals, leading to erroneous therapy discontinuation.

In our patient, EN occurred twenty days after the clinical manifestation of dermatophytosis, before oral administration of antifungals, avoiding misdiagnosis.

Griseofulvin was administered in all reported cases, comprising the present patient. Topical antimycotics were added in 53%, leading to regression of both EN and KC, highlighting the importance of recognizing the link between the two entities, to provide a correct combined diagnosis of both skin conditions and a sole efficacious therapeutic approach.

## Financial support

None declared.

## Authors’ contributions

Astrid Herzum: Study concept and design; Interpretation of data; statistical analysis; writing of the manuscript; effective participation in the research guidance; critical review of the literature; final approval of the final version of the manuscript.

Ehab Garibeh: Study concept and design; interpretation of data; writing of the manuscript; effective participation in the research guidance; critical review of the literature; final approval of the final version of the manuscript.

Lodovica Gariazzo: Study concept and design; interpretation of data; writing of the manuscript; effective participation in the research guidance; final approval of the final version of the manuscript.

Corrado Occella: Study concept and design; interpretation of data; writing of the manuscript; effective participation in the research guidance; final approval of the final version of the manuscript.

Gianmaria Viglizzo: Study concept and design; interpretation of data; writing of the manuscript; effective participation in the research guidance; final approval of the final version of the manuscript.

## Conflicts of interest

None declared.
